# A Novel Scheme for MIMO-SAR Systems Using Rotational Orbital Angular Momentum

**DOI:** 10.3390/s18103511

**Published:** 2018-10-18

**Authors:** Xiangxi Bu, Zhuo Zhang, Xingdong Liang, Longyong Chen, Haibo Tang, Zheng Zeng, Jie Wang

**Affiliations:** 1 Science and Technology on Microwave Imaging Laboratory, Institute of Electronics, Chinese Academy of Sciences, Beijing 100190, China; buxiangxi14@mails.ucas.edu.cn (X.B.); zhangzhuo@mail.ie.ac.cn (Z.Z.); lychen@mail.ie.ac.cn (L.C.); tanghaibo@mail.ie.ac.cn (H.T.); zengzheng16@mails.ucas.ac.cn (Z.Z.); 2 School of Electronics, Electrical and Communication Engineering, University of Chinese Academy of Sciences, Beijing 100049, China; 3 School of Electronic & Information Engineering, Nanjing University of Information Science & Technology, Nanjing 210044, China

**Keywords:** vortex electromagnetic (EM) waves, orbital angular momentum (OAM), multi-input–multi-output synthetic aperture radar (MIMO-SAR), Rotational Doppler Effect (RDE)

## Abstract

The vortex electromagnetic (EM) wave with orbital angular momentum (OAM) brings a new degree of freedom for synthetic aperture radar (SAR) imaging, although to date, its application to multi-input multi-output (MIMO) SAR has not yet been widely reported. In this paper, an orbital angular momentum (OAM)-based MIMO-SAR system is proposed. The rotational Doppler Effect (RDE) of vortex EM waves offers a novel scheme for an OAM-based MIMO-SAR system. By transmitting the rotational vortex EM waves, echoes of different OAM modes can be discriminated by a bandpass filter in the range-Doppler domain. The performance of the proposed scheme is independent of the time-variant channel responses, and the wider beam width of the vortex EM waves delivers, for the same antenna aperture size, better performance in terms of swath width and azimuth resolution, in contrast to the plane EM waves. Moreover, the spatial diversity of vortex EM waves shows great potential to enhance the MIMO-SAR system applications, which involve high-resolution wide-swath remote sensing, 3-D imaging, and radar-communication integration. The proposed scheme is verified by proof-of-concept experiments. This work presents a new application of vortex EM waves, which facilitates the development of new-generation and forthcoming SAR systems.

## 1. Introduction

As a novel radar system, the multi-input multi-output (MIMO) synthetic aperture radar (SAR) system, which combines the advantages of both MIMO and SAR technologies, has come to the forefront in recent years [1]. In MIMO-SAR systems, the equivalent observation channels can be greatly increased by using multiple transmitters and receivers [2]. The first investigation of MIMO-SAR emerged in 2006, which showed the advantages of MIMO-SAR systems ranging from high-resolution wide-swath remote sensing to multi-baseline interferometry, due to the increased equivalent observation channels [3]. Besides, MIMO-SAR can be exploited to improve the range resolution with efficient cross-track constellation configurations [4]. Moreover, MIMO-SAR can also be used in fully polarimetric SAR imaging to map a wider image swath without performance degradation [5]. To fully exploit the MIMO-SAR advantages mentioned above, it is necessary to separate within the receiver the scattered radar echoes from the multiple transmit signals, which is therefore a fundamental challenge for the implementation of MIMO-SAR systems [6]. Considering the ambiguity function introduced by Woodward [7], the energy of the imperfect orthogonal waveforms is spread but not removed in the time–frequency plane. For the distributed target with numerous closely spaced scatterers, the ambiguous energy from each scatterer will be accumulated, and thus the background noise level in both range and azimuth directions will be increased. Consequently, the imperfect orthogonal waveforms will deteriorate the MIMO SAR image quality dramatically [8]. Orthogonal waveforms designed in merely one dimension cannot avoid the ambiguous energy effectively. From the viewpoint of multidimensional modulation, orbital angular momentum (OAM) carried by vortex electromagnetic (EM) waves is introduced for MIMO-SAR implementations in this paper.

Independent of frequency and polarization, OAM has been widely studied in many realms; for example, the improvement of the spectrum efficiency in wireless communication [9], stealth target detection [10], and the super-resolution of radar imaging [11]. Recently, studies have shown that vortex EM waves of different OAM modes can be utilized in SAR imaging with great performance, and OAM presents a brand new degree of freedom (DOF) in SAR [12]. As a unique merit of vortex EM waves, the Rotational Doppler Effect (RDE) is associated with a frequency shift that is proportional to the product of the relative rotational angular frequency and the OAM mode [13]. The RDE can be used, not only to detect the spinning object [14], but also to distinguish each signal of various OAM modes [15,16]. The RDE of vortex EM waves brings a novel scheme for MIMO-SAR systems. By transmitting the rotational OAM beams, the transmitted signals of different OAM modes are fixed into specific center frequencies in the azimuth. Therefore, the echo signals of different OAM modes can be separated without ambiguities in the range–Doppler domain using bandpass filtering. Since the orthogonal separation is realized by filtering rather than decoding, the performance of the proposed scheme can avoid the impacts of the time-variant channel responses [7]. Besides, compared with plane EM waves, vortex EM waves have a wider beam width, which brings better performance in terms of swath width and azimuth resolution under the same antenna aperture size [12]. Based on the added DOF, the OAM-based scheme may greatly enhance the performance of MIMO-SAR applications, including high-resolution wide-swath remote sensing, 3-D imaging [17], stealth target detection [10], and radar-communication integration [18].

To our knowledge, there exist no public papers concerning the OAM-based MIMO-SAR system. In this article, a novel scheme for MIMO radar systems using the rotational OAM is proposed. Different OAM signals are mapped into different frequency shifts at the receiving end, and thus the orthogonal and spiral properties of OAM beams can be utilized in MIMO-SAR systems. The rest of this paper is organized as follows. In Section 2, the OAM-based MIMO-SAR system model is established. In Section 3, a novel OAM-based MIMO-SAR scheme using RDE is proposed, together with the processing progress. In Section 4, proof-of-concept experiments are presented to verify the scheme. In Section 5, a discussion is given. Finally, in Section 6, a conclusion of this work is provided.

## 2. An OAM-Based MIMO-SAR System Model

**Figure 1:**
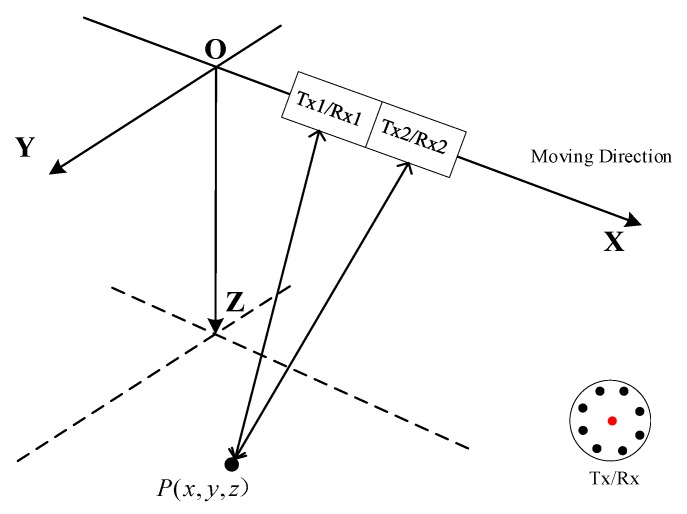
Geometry of the orbital angular momentum (OAM)-based multi-input multi-output (MIMO)-SAR system.

The geometry of OAM-based MIMO-SAR system under a platform coordinate is depicted in Figure 1. The system, which is working in stripmap mode, moves in the X-direction with velocity *v*. Assuming that there is an ideal point target P(x,y,z) in the detection area, for simplicity, a 2 × 2 MIMO-SAR system with two uniform circular array (UCA) antennas mounted in the along-track direction is discussed in this paper. The UCA antennas, as shown in the lower right corner of Figure 1, is used to transmit vortex EM waves of different OAM modes [19]. In the UCA antenna, the *N* array elements are located equidistantly around the circle, and each element is fed by a different phase, i.e., $\varphi_{n}=2\pi ln/N\;(n=1,2\ldots N)$, where *l* is the OAM mode number, and *n* is the element number. A single antenna (the red dot) set at the array center is used to receive the echoes. 

**Figure 2:**
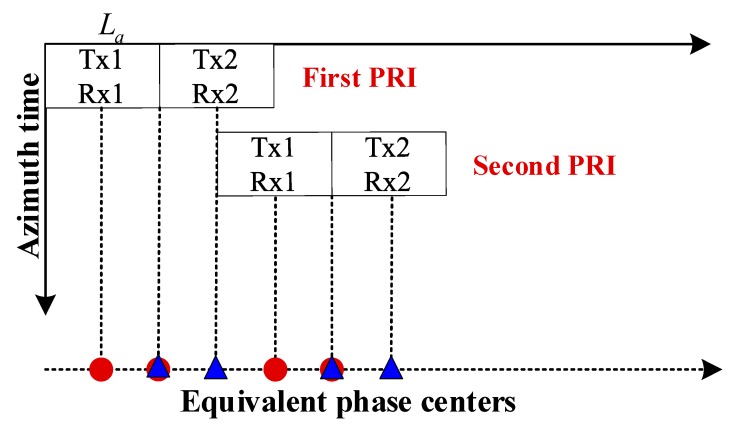
Distribution of the equivalent phase centers in the OAM-based MIMO-SAR system.

Figure 2 presents the distribution of the azimuth phase centers of the OAM-based MIMO-SAR system. The vertical axis denotes different azimuth times, and the horizontal axis denotes different equivalent phase centers. At every azimuth time, each receiving antenna can receive echoes from both Tx1 and Tx2. The red dot denotes the equivalent phase center of Tx1/Rx1 or Tx2/Rx1, while the blue triangle denotes the equivalent phase center of Tx1/Rx2 or Tx2/Rx2. There exist three equivalent observation channels at every azimuth time. In the case of SAR imaging, a wider image swath width can be obtained, due to a reduction of the pulse repetition frequency (PRF) by a factor of three compared to a single-channel system. Besides, the additional phase centers yield extra and longer baselines for the ground moving target indication (GMTI) and along-track interferometry (ATI) applications [2]. Moreover, in contrast to plane EM waves, vortex EM waves possess a wider beam width in both range and azimuth directions. Consequently, the OAM-based MIMO-SAR system can simultaneously realize the high azimuth resolution and the wide swath width with better performance. 

Assume that the OAM mode is pure, and that each element of the UCA antenna transmits the Linear Frequency Modulation (LFM) signal. The center of the 2 × 2 array is located at the origin when the azimuth time $t_{m}=0$, and the target azimuth position is $x-\left(p-1.5\right)L_{a}$ with respect to the *p*th (*p* = 1, 2) UCA antenna. According to Ref. [12], the signal transmitted from the *p*th Tx antenna can be expressed as:

(1) $$\begin{array}{c} s_{p}\left(t_{m},t,l_{p}\right)\approx N\exp\left\{il_{p}\pi /2\right\}J_{l_{p}}\left[ka\sin\theta_{p}\left(t_{m}\right)\right]\omega_{a}\left[t_{m}-x/v+(p-1.5)L_{a}/v\right]\omega_{r}\left[t-r_{p}\left(t_{m}\right)/c\right]\\ \exp\left\{i\pi K_{r}{\left[t-r_{p}\left(t_{m}\right)/c\right]}^{2}\right\}\exp\left\{i2\pi f_{c}\left[t-r_{p}\left(t_{m}\right)/c\right]\right\}\exp\left\{il_{p}\varphi_{p}\left(t_{m}\right)\right\} \end{array}$$

where t is the range time variable; $t_{m}$ is the azimuth time variable; $\omega_{r}(t)$ is the range envelope; $\omega_{a}(t_{m})$ is the azimuth envelope; v is the platform speed; $L_{a}$ is the aperture size of each UCA antenna; $K_{r}$ is the LFM ratio; $f_{c}$ is the central frequency of the signal; c is the speed of light; $l_{p}$ is the mode number of OAM beams transmitted by the *p*th UCA antenna; $\varphi_{p}\left(t_{m}\right)$ is the target azimuth angle history, which can be expressed as:

(2) $$\varphi_{p}(t_{m})=\tan^{-1}\left\{\frac{y\cos\beta -z\sin\beta }{x-vt_{m}-\left(p-1.5\right)L_{a}}\right\};$$

where β is the antenna look-down angle; $J_{l}\left[ka\sin\theta (t_{m})\right]$ denotes the *l*th order Bessel function of the first kind; *k* is the wave number; *a* is the radius of the UCA antenna; $\theta_{p}(t_{m})$ is the target polar angle history, which can be expressed as:

(3) $$\theta_{p}(t_{m})=\sin^{-1}\left\{\frac{\sqrt{{\left(x-vt_{m}-\left(p-1.5\right)L_{a}\right)}^{2}+{\left(y\cos\beta -z\sin\beta \right)}^{2}}}{\sqrt{y^{2}+z^{2}+{\left(x-vt_{m}-\left(p-1.5\right)L_{a}\right)}^{2}}}\right\}$$

where $r_{p}\left(t_{m}\right)$ is the target range history, which can be expressed as:

(4) $$r_{p}(t_{m})=\sqrt{{\left(x-vt_{m}-\left(p-1.5\right)L_{a}\right)}^{2}+y^{2}+z^{2}}.$$

The target azimuth position with respect to the *p*th (*p* = 1, 2) Tx antenna and the *q*th (*q* = 1, 2) Rx antenna is $x-\left(p+q-3\right)L_{a}/2$, and the received signal $s_{p,q}$ can be expressed as:

(5) $$\begin{array}{c} s_{p,q}\left(t_{m},t,l_{p}\right)\approx N\exp\left\{il_{p}\pi /2\right\}J_{l_{p}}\left[ka\sin\theta_{p}\left(t_{m}\right)\right]\omega_{a}\left[t_{m}-x/v+(p+q-3)L_{a}/2v\right]\omega_{r}\left[t-2r_{p,q}\left(t_{m}\right)/c\right]\\ \exp\left\{i\pi K_{r}{\left[t-2r_{p,q}\left(t_{m}\right)/c\right]}^{2}\right\}\exp\left\{i2\pi f_{c}\left[t-2r_{p,q}\left(t_{m}\right)/c\right]\right\}\exp\left\{il_{p}\varphi_{p}\left(t_{m}\right)\right\} \end{array}$$

where the target azimuth position is $x-\left(q-1.5\right)L_{a}$ with respect to the *q*th Rx antenna, and $r_{p,q}\left(t_{m}\right)$ is the round trip target range history, which can be expressed as:

(6) $$r_{p,q}(t_{m})=\sqrt{{\left(x-vt_{m}-\left(p-1.5\right)L_{a}\right)}^{2}+y^{2}+z^{2}}+\sqrt{{\left(x-vt_{m}-\left(q-1.5\right)L_{a}\right)}^{2}+y^{2}+z^{2}}.$$

For a certain receiving antenna, the superimposed echo signals radiated by both Tx1 and Tx2 can be received. Under this circumstance, the orthogonal properties of OAM cannot be utilized, so that it is hard to distinguish each echo signal of the different OAM modes.

## 3. A Novel RDE-Based Scheme

To solve the problem mentioned above, a novel RDE-based scheme used for the MIMO-SAR system is proposed in this section. 

The rotational Doppler Effect (RDE) occurs when relative rotation between a vortex EM wave source and an observer exists [20]. For the linear polarized vortex EM waves of the OAM mode *l*, the rotational Doppler frequency shift Δf is:

(7) $$\Delta f=\frac{l\Omega }{2\pi }$$

where Ω is the relative rotation speed between the source and the observer. 

If the phase of each element in UCA antenna varies azimuthally, i.e., the *n*th element is fed by $\varphi_{n-1}=2\pi l(n-1)/N$ after one period of τ, the rotational OAM beams can be generated, and the equivalent rotation frequency is 1/Nτ r/s. When the UCA antennas in Figure 1 transmit the rotational OAM beams, Equation (5) can be rewritten as:

(8) $$\begin{array}{c} s_{p,q}\left(t_{m},t,l_{p}\right)\approx N\exp\left\{il_{p}\pi /2\right\}J_{l_{p}}\left[ka\sin\theta_{p}\left(t_{m}\right)\right]\omega_{a}\left[t_{m}-x/v+(p+q-3)L_{a}/2v\right]\omega_{r}\left[t-2r_{p,q}\left(t_{m}\right)/c\right]\\ \exp\left\{i\pi K_{r}{\left[t-2r_{p,q}\left(t_{m}\right)/c\right]}^{2}\right\}\exp\left\{i2\pi f_{c}\left[t-2r_{p,q}\left(t_{m}\right)/c\right]\right\}\exp\left\{il_{p}\left[\Omega_{p}t_{m}+\varphi_{p}\left(t_{m}\right)\right]\right\} \end{array}$$

where $\Omega_{p}$ is the rotation speed of the *p*th OAM beams. 

The signal received by the *q*th (*q* = 1, 2) antenna is:

(9) $$s_{q}\left(t_{m},t\right)=s_{1}{}^{\prime }\left(t_{m},t\right)\exp\left\{il_{1}\Omega_{1}t_{m}\right\}+s_{2}{}^{\prime }\left(t_{m},t\right)\exp\left\{il_{2}\Omega_{2}t_{m}\right\}$$

where:

(10) $$\begin{array}{c} s_{1}{}^{\prime }\left(t_{m},t\right)=N\exp\left\{il_{1}\pi /2\right\}J_{l_{1}}\left[ka\sin\theta_{1}\left(t_{m}\right)\right]\omega_{a}\left[t_{m}-x/v+(q-2)L_{a}/2v\right]\omega_{r}\left[t-2r_{1,q}\left(t_{m}\right)/c\right]\\ \exp\left\{i\pi K_{r}{\left[t-2r_{1,q}\left(t_{m}\right)/c\right]}^{2}\right\}\exp\left\{i2\pi f_{c}\left[t-2r_{1,q}\left(t_{m}\right)/c\right]\right\}\exp\left\{il_{1}\varphi_{1}\left(t_{m}\right)\right\}, \end{array}$$

and:

(11) $$\begin{array}{c} s_{2}{}^{\prime }\left(t_{m},t\right)=N\exp\left\{il_{2}\pi /2\right\}J_{l_{2}}\left[ka\sin\theta_{2}\left(t_{m}\right)\right]\omega_{a}\left[t_{m}-x/v+(q-1)L_{a}/2v\right]\omega_{r}\left[t-2r_{2,q}\left(t_{m}\right)/c\right]\\ \exp\left\{i\pi K_{r}{\left[t-2r_{2,q}\left(t_{m}\right)/c\right]}^{2}\right\}\exp\left\{i2\pi f_{c}\left[t-2r_{2,q}\left(t_{m}\right)/c\right]\right\}\exp\left\{il_{2}\varphi_{2}\left(t_{m}\right)\right\}. \end{array}$$

After azimuth Fourier transforming, Equation (9) in the range-Doppler domain can be expressed by:

(12) $$S_{q}\left(f_{a},t\right)=W\left(f_{a}-\frac{l_{1}\Omega_{1}}{2\pi }\right)S_{1}\left(f_{a},t\right)+W\left(f_{a}-\frac{l_{2}\Omega_{2}}{2\pi }\right)S_{2}\left(f_{a},t\right)$$

where $f_{a}$ is the azimuth frequency.

It can be seen from Equation (12) that, due to the RDE, the echo signals related to the different OAM modes locate at different azimuth frequencies. Assume that $\Omega_{1}$ is 0 rad/s and $l_{2}\Omega_{2}/2\pi$ is PRF/2; Equation (12) can be rewritten as:

(13) $$S_{q}\left(f_{a},t\right)=W\left(f_{a}\right)S_{1}\left(f_{a},t\right)+W\left(f_{a}-\frac{\mathrm{PRF}}{2}\right)S_{2}\left(f_{a},t\right).$$

**Figure 3:**
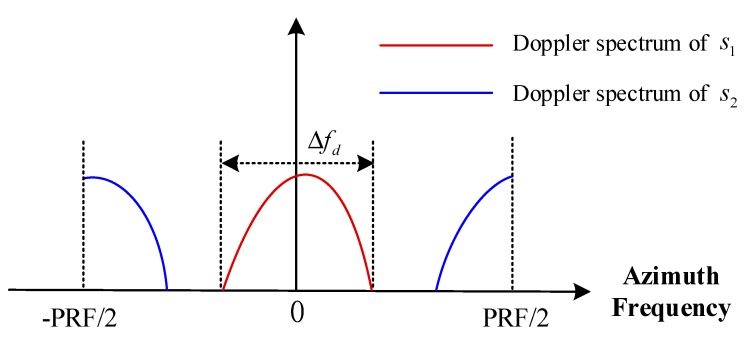
Doppler frequency spectrum of the received signal.

To better understand this process, Figure 3 shows the Doppler spectrum of the received signal. The red line denotes the spectrum of $S_{1}$, which locates at $f_{a}=0$, while the blue line denotes the spectrum of $S_{2}$, which locates at $f_{a}=\mathrm{PRF}/2$. Each spectrum has the same Doppler bandwidth $\Delta f_{d}$.

In the case of aliasing in the Doppler domain, PRF should satisfy:

(14) $$\mathrm{PRF}>2\Delta f_{d}$$

**Figure 4:**
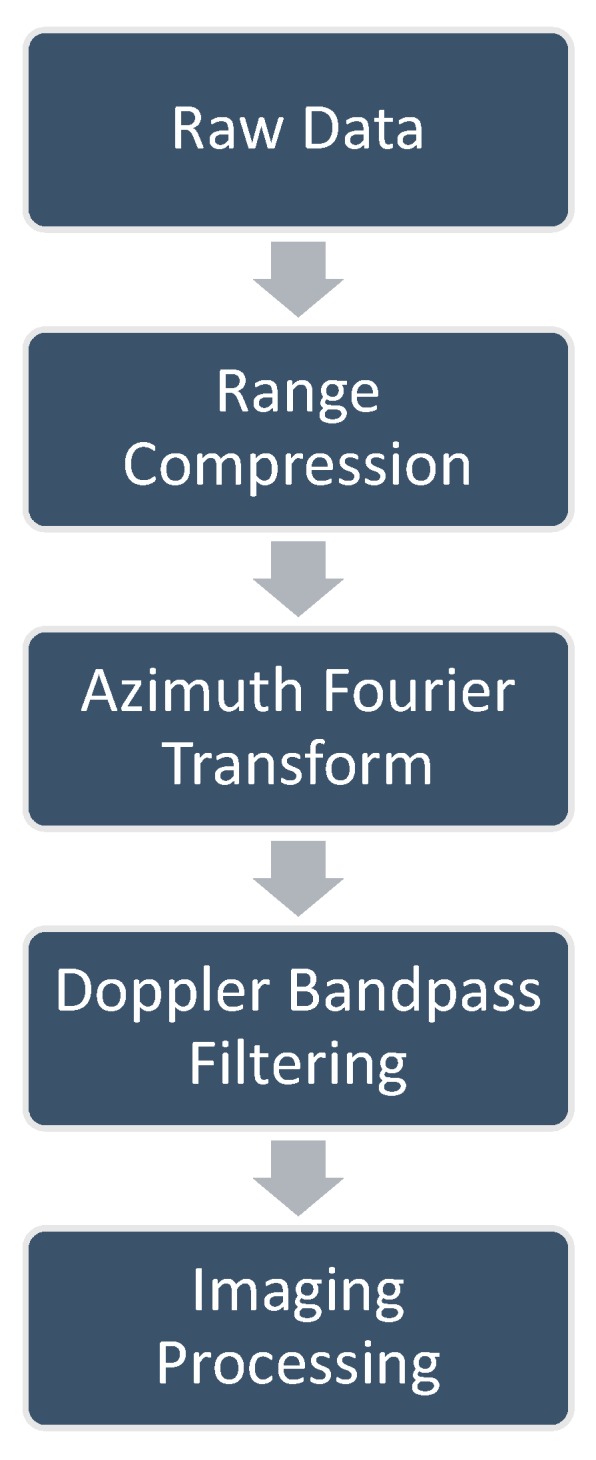
Processing flowchart of the OAM-based MIMO-SAR data.

As shown in Figure 4, for a given receiver, the OAM-based MIMO-SAR data can be processed by the following steps:

(1) Range compression of the raw data;
(2) Azimuth Fourier transform of the data;
(3) Separating the data of different OAM modes in the range–Doppler domain by bandpass filtering;
(4) Imaging processing to obtain the image. The 2-D SAR image can be obtained by the classic SAR imaging algorithm, e.g., the Range–Doppler algorithm or the ωK algorithm [12].

Compared with the conventional orthogonal waveform schemes, the data processing procedure of our proposed scheme was simpler, which could avoid some complicated demodulation steps.

In our scheme, the rotational Doppler shift was implemented by the virtual rotation movement of the antenna, which enabled the frequency division multiplexing technique in the Doppler domain. The translational Doppler shift was still implemented by the movement of the SAR platform, which was used for virtual synthetic aperture in the azimuth.

Due to the orthogonality of the vortex EM waves, the intra-modal interference could be suppressed in our proposed OAM-based MIMO-SAR system, and it became applicable for one single system to simultaneously perform high-resolution SAR imaging and wireless communication in the same frequency band. For example, in our scheme, one OAM mode could be used for high-resolution SAR imaging, and because of the added equivalent phase centers and wider beam width, the swath width could be twice that of the SISO mode without range ambiguity. Meanwhile, the other OAM mode could be utilized for wireless communication with better performance [21]. Moreover, because our scheme was implemented by bandpass filtering in the Doppler domain rather than decoding, the performance of our scheme could escape from the impacts of the time-variant channel responses [7].

## 4. Results

**Figure 5:**
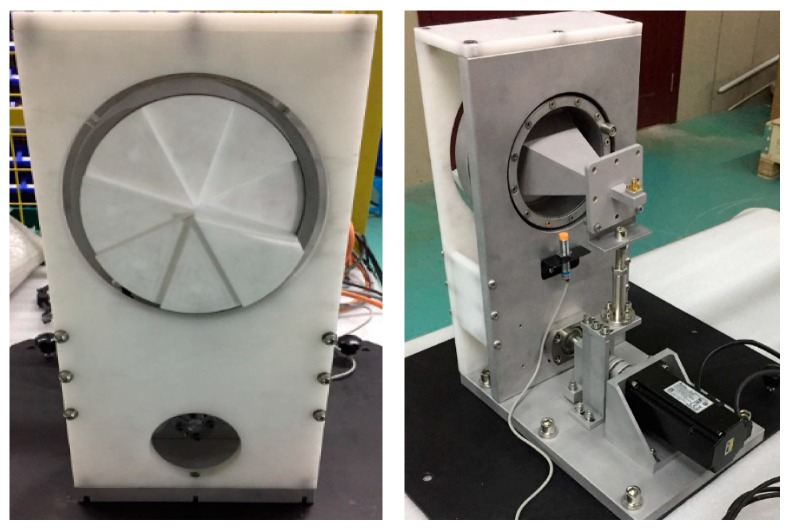
Prototypes of the transmitting antenna platform.

In this section, we present some experiments to verify our proposed scheme at the proof-of-concept level. RDE experiments, referred to as single-in–single-out (SISO) and multiple-in–single-out (MISO) were carried out. The prototype of the transmitting antenna platform, which can transmit the rotational OAM beams, is shown in Figure 5. The OAM beams were generated by putting a spiral phase plate (SPP) ahead of a horn antenna with 20 dB gain [22]. In our experiments, two SPPs were used, i.e., mode +1 and mode −1. Before the experiments, the radiation pattern of the transmitting antenna was measured to make sure that they could generate vortex EM waves of correct OAM modes. The electrical machinery was used to rotate the SPP mechanically, and the rotational speed could be adjusted by the controller. Without changing the polarization, the rotational OAM beams could be generated by the platform.

### 4.1. SISO Mode

**Figure 6:**
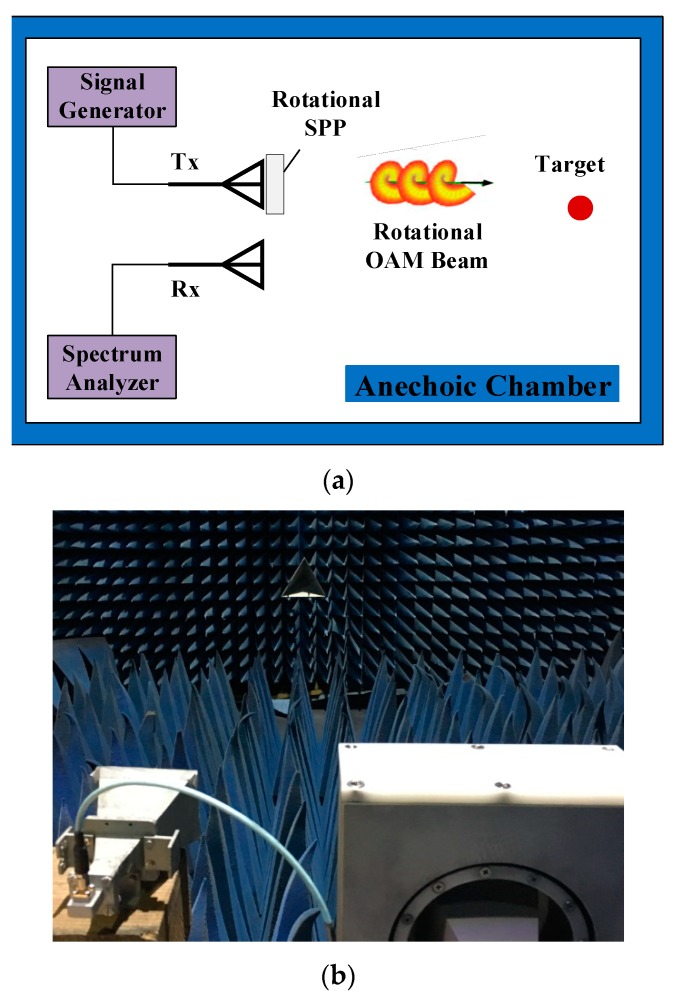
(**a**) Schematic configuration of the single-in–single-out (SISO) mode; (**b**) experimental scenario of the SISO mode.

The schematic configuration of the SISO mode is shown in Figure 6a, and the experimental scenario is displayed in Figure 6b. In the SISO mode, the rotational OAM beams were generated by the transmitting antenna platform connected to the signal generator. At the receiving end, another horn antenna with 20 dB gain was used to receive the backscattered signal from the target, and a spectrum analyzer was used to observe the frequency spectrum. The polarizations of both Tx and Rx were the same. The target distance was 3 m, which satisfied the far-field condition. The signal generator generated a continuous wave (CW) signal at 14.5 GHz.

**Figure 7:**
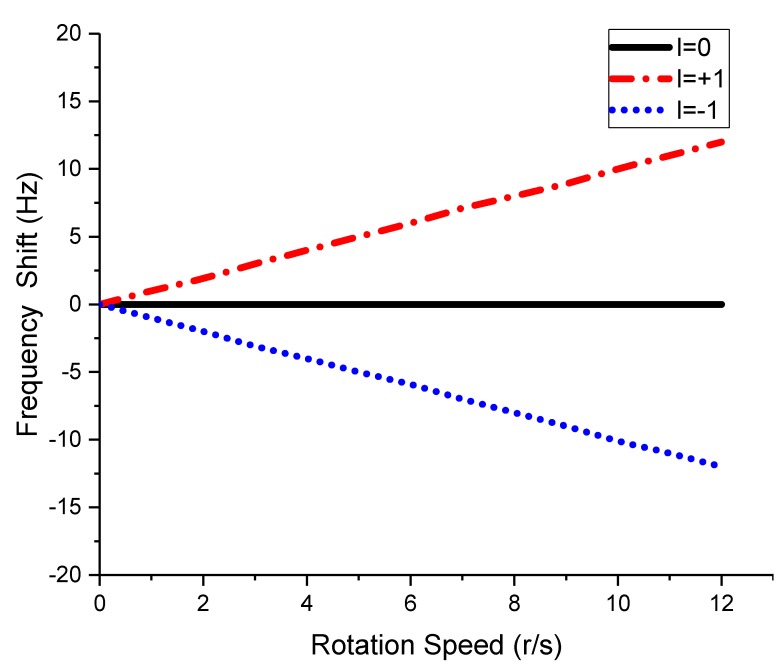
The rotational Doppler frequency shifts of different OAM echoes versus the rotation speed.

The rotational Doppler frequency shifts of different OAM echoes versus the rotation speed are shown in Figure 7. For conventional plane EM waves (*l* = 0), there existed no frequency shift, whether the incident wave rotated or not. For vortex EM waves, the frequency shifts of the two OAM modes, i.e., *l* = +1 and *l* = −1, were different. The frequency shift also increased with the rotation speed. The experimental results showed that with the rotational vortex EM waves, a different OAM mode brought a different frequency shift at the receiving end, which made it possible to distinguish different OAM modes in the frequency domain. 

### 4.2. MISO Mode

**Figure 8:**
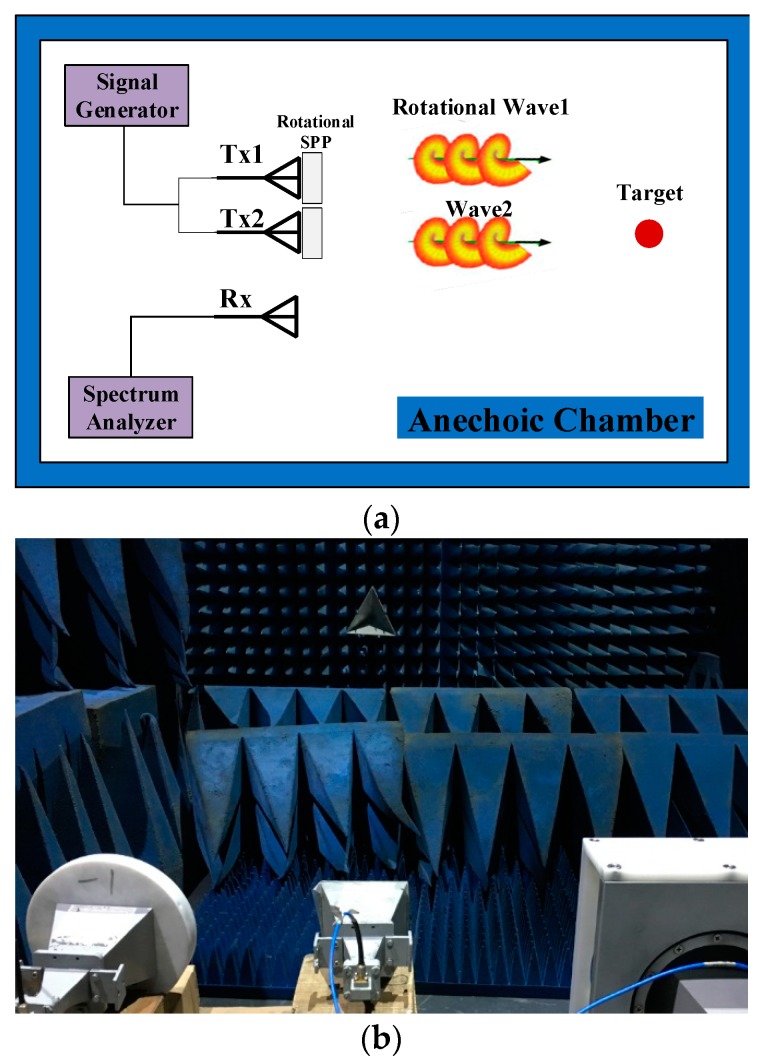
(**a**) Schematic configuration of the MISO mode; (**b**) experimental scenario of the MISO mode.

To further verify the effectiveness of our scheme, an experiment on the MISO mode was conducted. As shown in Figure 8, most of the configurations were the same as those in the SISO mode, except for the transmitting end. The signal from the signal generator was divided into two signals by the power divider. One connected to Tx1 transmitted the rotational vortex EM waves of mode +1, and the other one connected to Tx2 transmitted vortex EM waves of mode −1. The horn antenna in the middle was still used to receive the superimposed signals from the target. The center frequency of the transmitting CW signal was 14.5 GHz. 

**Figure 9:**
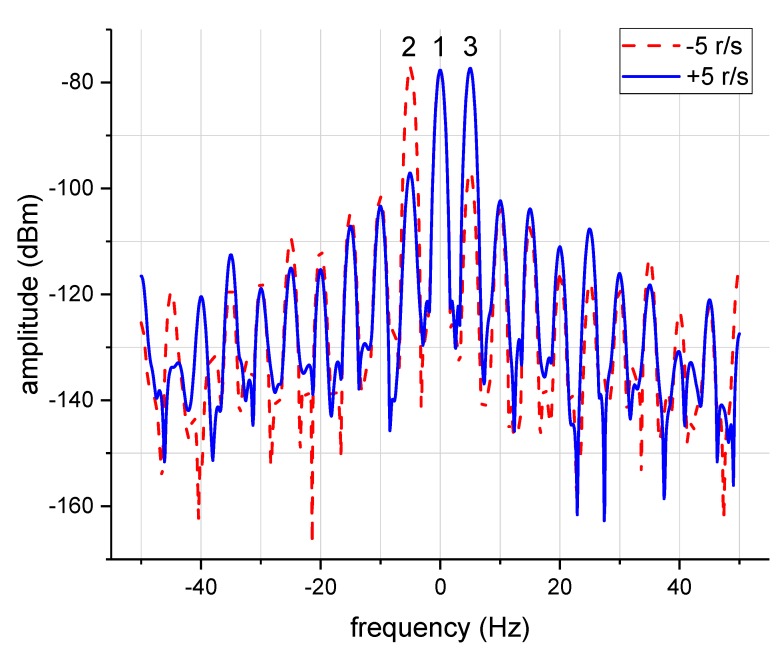
The frequency spectrums of the received signals for the rotation speed is −5 r/s (dash red), and the rotation speed is +5 r/s (solid blue).

The spectrums of the received signals are shown in Figure 9. The red line denotes the spectrum when the rotation speed is 5 r/s in an anti-clockwise direction. Marker1, which denotes the echo of stationary mode −1, was located at 14.5 GHz (0 Hz in Figure 9), while Marker2 was shifted −5 Hz from Marker1, which denoted the echo of rotational mode +1. When the rotation direction was changed to a clockwise direction, a +5 Hz frequency shift was obtained between Marker1 and Marker3, as shown in Figure 9 (the blue line). In contrast, the different frequency shifts among the two cases indicated that the rotational Doppler shift was determined by not only the rotation speed, but also the rotation direction, which was consistent with the theory. Consequently, the echoes of different OAM modes could be easily separated by the bandpass filter in the frequency domain. 

## 5. Discussion

Based on RDE, a novel scheme of the OAM-based MIMO-SAR system is proposed in this paper. In Section 2, a simplified 2 × 2 model is established, which is a MIMO-SAR with two transmit and two receive channels that together provide three virtual phase centers. More antennas can be used to increase the number of observation channels, which could further enhance the performance of the OAM-based MIMO-SAR system. 

The proposed scheme is verified at a proof-of-concept level, through the RDE experiments referred to SISO and MISO modes. In the experiments, the rotational OAM beam was implemented by rotating the SPP mechanically, and thus the rotation speed cannot be very high, which limits the rotational Doppler frequency shift. The virtually rotational UCA antenna scheme introduced in Section 3 is a more suitable way to generate the desired rotational OAM beams. 

Note that in Figure 9, apart from two main lobes, other harmonic frequency components also exist, which indicates that the transmitted OAM beam from SPP is not of pure OAM mode. The main OAM mode accounted for the highest proportion, while other OAM modes give some minor contributions, which can also be filtered in the frequency domain. 

## 6. Conclusions

In this article, a novel scheme of OAM-based MIMO-SAR systems using the rotational Doppler Effect is proposed for the first time. Based on the proposed scheme, the mathematical model is established, and the corresponding signal processing algorithm is presented. Moreover, proof-of-concept experiments are carried out in an anechoic chamber to verify the scheme. 

Much attention has been paid towards OAM-based wireless communication. The OAM-based MIMO system can bring a vast reduction in receiver complexity without a capacity penalty [21]. A new configuration of OAM-based MIMO communication system has been proposed, which has a better performance, in terms of capacity gain and bit-error-rate, than the conventional MIMO communication system [23]. The proposed OAM-based MIMO-SAR system, which simultaneously transmits mutually orthogonal vortex EM waves in the same frequency band, can acquire adequate degrees of freedom (DOF) to realize advanced radar-communication integration, which combines high-resolution wide-swath remote sensing, and wireless communication with better performance. 

This work is regarded as a first step in this direction. Our future work contains OAM antenna design to generate high quality OAM beams and a practical implementation of the OAM-based MIMO-SAR system.
